# Spinosin ameliorates insulin resistance by suppressing reactive oxygen species-associated inflammation

**DOI:** 10.22038/IJBMS.2022.64154.14127

**Published:** 2022-07

**Authors:** Chi-yu Ge, Ling Yang, Jun-li Zhang, Zhi-feng Wei, Feng Feng

**Affiliations:** 1 School of Pharmacy, Jiangsu Food and Pharmaceutical Science College, Huai’an, Jiangsu 223005, China; 2 Jiangsu Protein Drug Engineering Lab, Huai’an, Jiangsu 223005, China; 3 Department of Pharmacology of Chinese Materia Medica, School of Traditional Chinese Pharmacy, China Pharmaceutical University, 24 Tong Jia Xiang, Nanjing 210009, China

**Keywords:** Inflammation, Insulin receptor substrate – proteins, Insulin resistance, Reactive oxygen species, Spinosin, Zizyphus

## Abstract

**Objective(s):**

Spinosin is the predominant C-glycoside flavonoid derived from the seeds of *Zizyphus jujuba *var. Spinosa (Rhamnaceae). The present study aimed to investigate the effects of spinosin on insulin resistance (IR) in vascular endothelium.

**Materials and Methods:**

The anti-IR effect of spinosin was evaluated in a high-fat diet (HFD) treated mice model. The effects of spinosin pretreatment on reactive oxygen species (ROS)-associated inflammation in Human umbilical vein endothelial cells (HUVEC) were evaluated by western blot analysis and reverse transcription-polymerase chain reaction. The effect of spinosin on insulin-mediated endothelium-dependent vasodilation of rat aortae was further evaluated.

**Results:**

Spinosin at 20 mg/kg alleviates increased mice’s body weight, fasting serum glucose, oral glucose tolerance, serum insulin, insulin resistance index, and serum lipid of HFD-treated mice. Spinosin at 20 μM suppressed ROS overproduction, and inhibited ROS-related HUVEC inflammation by inhibiting mRNA expression of tumor necrosis factor-α and interleukin-6. In addition, spinosin at 0.1 μM showed a vasodilation effect of isoprenaline-pretreated rat aortae and increased insulin-mediated NO production in endothelial cells. These effects were shown to be related to the spinosin regulating serine/tyrosine phosphorylation of insulin receptor substrate-1 (IRS-1) facilitated/phosphoinositide 3-kinase (PI3K) signaling.

**Conclusion:**

Spinosin may ameliorate IR and ROS-associated inflammation, and increase endothelial NO production by mediating IRS-1/PI3K/endothelial nitric oxide synthase (eNOS) pathway.

## Introduction

Diabetes mellitus (DM) is one of the most frequent chronic diseases characterized by multisystem complications (1). In 2018, the global prevalence of diabetes had risen from 4.7% in 1980 to 12.8% (2). The leading cause of morbidity and mortality of diabetes is attributable to its vascular complications, and endothelial insulin resistance (IR) plays a major role in the development of vascular complications. IR can induce endothelial dysfunction by reduced NO bioavailability, increased oxidative stress, and elevated expression of proinflammatory factors (3-4), and protection of endothelial IR is becoming critical for the prevention and improvement of vascular complications of DM. Reactive oxygen species (ROS) associated insulin receptor substrate (IRS)/phosphoinositide 3-kinase (PI3K)/endothelial nitric oxide synthase (eNOS) pathway play key roles in the occurrence of IR induced endothelial dysfunction. Although hyperglycemia is recognized as the primary factor in the pathogenesis of diabetic complications (5), evidence indicates that excessive free fatty acids (FFA) in circulation lead to ROS overproduction in the early stages of diabetes. During this time, ROS-associated inflammation triggering the activation/inhibition of signaling cascades that cause IR, notably, tumor necrosis factor α (TNF-α) and interleukin- 6 (IL-6), can affect IRS-1 function and impair insulin signaling (6). Meanwhile, inflammation and ROS can activate mitogen-activated protein kinases (MAPKs), which promote adipocyte inflammation and IR by modulation of IRS-1 phosphorylation (7,8). The negative regulation of IRS-1 phosphorylation thereby leads to the deficiencies of endothelial-derived NO by downstream signaling along the PI3K/Akt/eNOS pathway, which further leads to endothelium-dependent vasodilation.

Spinosin is a C-glycoside flavonoid isolated from the seeds of *Zizyphus jujuba* var. Spinosa. In view of its effectiveness and safety, spinosin is recognized as a marker compound for quality control of *Z. jujuba* (9-11). Notably, *Z. jujuba* or spinosin showed both anti-inflammatory and antioxidant effects (12-14). Therefore, we wanted to know whether these actions contributed to the amelioration of endothelial dysfunction in IR. The present study aimed to investigate the effects of spinosin on improving NO production in endothelium by focusing on the pathways involved in ROS-associated inflammation and endothelial IR. 

## Materials and Methods

***Materials***

The chemical reagents, ELISA kits, and polyclonal antibodies used in this work were listed in the supplementary materials.

***Establishment of IR model in mice***

Sixty male ICR (Institue of Cancer Research) mice aged 4–5 weeks, 18–20 g, were provided by Qinglongshan animal breeding farm (Nanjing, China) [Production license No. SCXK (Su) 2017-0001]. The animals were fed and drank freely at 22±2 °C for 3 days before the experiment, and their body weight was measured every day. The animals were randomly divided into the normal group (standard chow diet, Chow) and the high-fat diet (HFD) group (HFD was purchased from Teluofei feed Technology Co., Ltd, Nantong, China); After feeding for two weeks, the high-fat diet group was randomly divided into the model group (HFD), spinosin (10 mg/kg) group, spinosin (20 mg/kg) group, spinosin (40 mg/kg) group, and metformin (200 mg/kg) group, both spinosin and metformin were intragastrically administrated with continued HFD. The mice in the chow group were fed a normal control diet (10% low-fat diet) for 4 weeks. Fasting body weight and food consumption were measured every week, fasting serum glucose level was measured after 4 weeks of gavage, and oral glucose tolerance test (OGTT) was performed. The homeostasis model of insulin resistance (HOMA-IR) is the gold standard for estimating IR and is derived using the following equation (15):

HOMA-IR=fasting serum glucose(mmol/L)×fasting serum insulin(mU/L)/22.5

Success induction of IR was evidenced by increased fasting serum insulin and HOMA-IR values compared with the chow group (15). FFA, total cholesterol (T-Cho), triglyceride (TG) and low-density lipoprotein cholesterol (LDL-C), and low levels of high-density lipoprotein cholesterol (HDL-C), ROS was measured by a test kit according to the manufacturer’s insruction; plasminogen activator inhibitor-1 (PAI-1) and high-sensitivity C-reactive protein (hs-CRP) human soluble intercellular adhesion molecule-1 (sICAM-1), IL-6, and TNF-a were measured by an ELISA kit according to manufacturer’s introduction (supplementary materials). All protocols were approved by the Ethics Committee of China Pharmaceutical University.

***Endothelial cell injury model induced by palmitic acid (PA) and insulin***

HUVECs were cultured in Dulbecco’s modified eagle’s medium (DMEM) with 10% fetal bovine serum and 1% penicillin and streptomycin at 37 °C and 5% CO_2_. Logarithmic growth phase cells were selected for experiments. 

PA-induced endothelial cell injury: HUVEC cells were cultured with spinosin (10, 20, and 40 μM) and 20 mM metformin for 30 min, followed by the addition of 100 μM PA for 24 hr. The cell culture supernatants were taken for subsequent experiments. PA and insulin-induced endothelial cell injury: HUVEC cells were cultured with spinosin (10, 20, and 40 μM) and 20 mM metformin (or LY-294002) for 30 min, followed by the addition of 100 μM PA for 24 hr and then 0.1 μM insulin for 30 min. The cell culture supernatants were taken for subsequent experiments. p38, ERK, JNK, IRS-1, PI3K, Akt, and eNOS levels were measured by western blot, and NO level was measured by an assay kit according to the manufacturer’s introduction (supplementary materials).

***Preparation of isolated intact endothelial and endothelial-derived rat aortae***

Carbon dioxide was used to euthanize male Sprague-Dawley rats (body weight: 250–300 g). The descending thoracic aortae were removed and bathed in Krebs-Henseleit (K-H) solution. Connective tissue and fat were dissected and removed from periaortic tissues under a microscope. The aortae were cut into aortic rings approximately 3-4 mm wide. These isolated aortic rings intact with endothelium were suspended in an organ bath containing 5 ml of K-H solution that was attached to an isolated tissue perfusion device and tension sensor (JH-2, Chengdu Taimeng Software Co., Ltd.). The preparation of endothelial-derived aortic rings was similar to that mentioned above, except the intact endothelial aortic ring was removed by inserting 25-gauge needles into the lumen of the isolated aortae and rolling the aortae back and forth for a few seconds.

The changes in rat aortae tension were recorded by BL-420s Biological Functional System (Chengdu Taimeng Software Co., Ltd.). A baseline resting tension of 2.0 g was maintained for 30 min to reach equilibrium, and the K-H solution was exchanged every 10 min. To confirm the physiological activity of the isolated aortae, KCl (3 M) was added to the organ bath containing the aortae, allowed to achieve equilibrium for 15 min, any concentration less than 0.5 g in response to KCl was considered to be damaged or necrotic, and the aortae were discarded. To evaluate the integrity of endothelium of isolated rat aortae, we added 5 μl isoprenaline (ISO) (1 mM) into the organ bath containing the aortae, allowed to achieve equilibrium for 8 min. After ISO produced a sustained and stable concentration, we sequentially added acetylcholine (10^-6^ to 10^-4^ M) into the organ bath, the aortae that relaxed by more than 75% in response to acetylcholine were considered to be of a complete endothelium, if the aortae were relaxed by less than 15%, indicating the complete denudation of its endothelium. After the aortic rings showing acetylcholine-induced relaxation were washed with fresh K-H solution 3 times, baseline resting tension was recovered.

***Vasodilation effect of spinosin on isolated rat aortae***

The contraction of isolated rat aortic rings was induced by adding 5 μl 1 mM ISO, and the magnitude of ISO-induced contraction was recorded. Next, the ISO solution was washed with fresh K-H solution, and baseline resting tension was recorded. Then 1 μl of 0.5 mM spinosin solution, 1, 9, and 99 μl 5 mM spinosin solutions were sequentially added into the water bath, and the spinosin of each concentration was added until the tension curve was stable. The corresponding relaxation rates were finally recorded.

***Effect of LY294002 on the vasodilation effect of spinosin to ISO induced aortic ring contraction***

After the integrity of the endothelium was examined by acetylcholine, the aortic rings were incubated in the K-H solution with added 5 μl 100 mm LY294002 (control group added K-H solution) for 30 min. 5 μl of 1mM ISO was added to the solution for aortic ring contraction. After the tension was stable, 1 μl 0.5 mM spinosin, 1, 9, and 99 μl 5 mM spinosyn were sequentially added into the water bath, and spinosin of each concentration was added until the tension curve was stable. The diastolic rate of the inhibitor group was compared with that of the control group.

***Data analysis***

All data were expressed as mean ± SEM. The statistical differences between groups were evaluated by ANOVA, and *P*<0.05 indicates significant difference.

## Results

***Spinosin alleviates increased mice body weight, fasting serum glucose, oral glucose tolerance, serum insulin, IR index, and serum lipids of HFD-treated mice***

**Figure 1:**
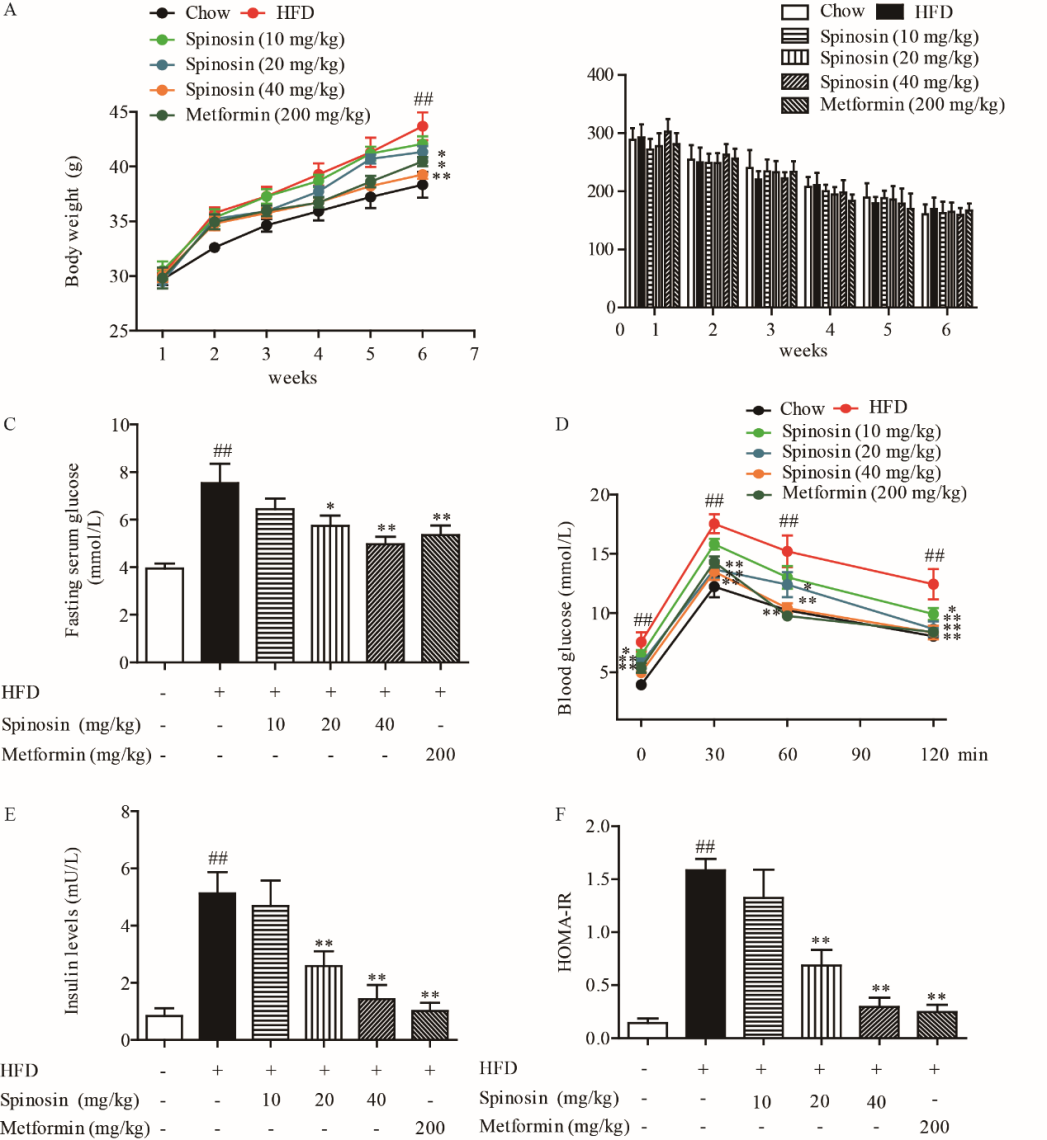
Effect of spinosin on body weight, food intake, fasting serum glucose, oral glucose tolerance, serum insulin, and insulin resistance index of high-fat diet (HFD)-treated mice. Mice were randomly divided into chow group, HFD group, spinosin (10, 20, 40 mg/kg) group, and metformin (200 mg/kg) group. (A) Body weight. (B) Food intake. (C) Fasting serum glucose (mmol/l). (D) Oral glucose tolerance test (OGTT) (mmol/l). (E) Insulin level in serum (mU/L). (F) Homeostasis model of assessment for insulin resistance index (HOMA-IR). All data were presented as mean ± SEM (n=7). ##*P*<0.01 vs chow group; *P<0.05 and **P<0.01 vs HFD group

IR was induced on ICR mice by feeding 60% HFD. As shown in Figure 1A, the weight of HFD group mice continued to increase. Intragastric administration of spinosin (20 and 40 mg/kg) and metformin (200 mg/kg) could significantly inhibit the trend of weight increase in mice. Compared with the HFD group, there was no significant difference in food intake between spinosin and metformin groups, indicating that the effect was not produced by reducing food consumption (Figure 1B). In addition, as shown in Figure 1C, the fasting serum glucose level in the HFD group was significantly increased, while spinosin (20 and 40 mg/kg) and metformin (200 mg/kg) significantly decreased the serum glucose level.

In OGTT, after intragastric administration of glucose, the serum glucose level of mice in each group increased first and then decreased, especially in the HFD group, which indicated that there was obviously impaired glucose tolerance (Figure 1D). Spinosin and metformin could resist the impaired glucose tolerance induced by HFD. In the HFD group, IR was observed which was evidenced by increased fasting serum insulin level and HOMA-IR values compared with the chow group (*P*<0.01, Figures 1E and F), which indicated the IR model was successfully induced. Spinosin (20 and 40 mg/kg) and metformin (200 mg/kg) could reduce the serum insulin level, down-regulate HOMA-IR, and improve IR. High levels of FFA, T-Cho, TG, LDL-C, and HDL-C are the early manifestations of IR (16)(Supplementary materials Figure S1A). We studied the effect of spinosyn on the serum levels of FFA, T-Cho, TG, LDL-C, and HDL-C of HFD-treated mice. As shown in Supplementary materials’ Figure S1A, the serum FFA level of the HFD group increased, while spinosin (20 and 40 mg/kg) and metformin (200 mg/kg) significantly decreased serum FFA. Meanwhile, the serum levels of T-Cho, TG, and LDL-C in the HFD group were significantly increased, while the serum levels of HDL-C were decreased. Spinosin and metformin could reduce the serum levels of T-Cho, TC, and LDL, but there was no significant improvement in HDL (Supplementary materials, Figure S1B-E).

***Spinosin alleviates increased PAI-1 and hs-CRP in HFD-treated mice***

PAI-1 is a single chain glycoprotein belonging to the serine protease inhibitor family. PAI-1 mainly comes from adipose tissue, vascular endothelium, and liver, and is an important inhibitor of plasminogen activation *in vivo*. PAI-1 level is positively correlated with fasting insulin level and HOMA-IR. As shown in Supplementary materials’ Figure S2A, the serum PAI-1 level in the HFD group was significantly increased and significantly decreased in both spinosin (20 and 40 mg/kg) and metformin (200 mg/kg) groups. As a non-specific inflammatory marker, hs-CRP is an acute-phase reactive protein after body injury or inflammatory reaction. When CRP is at a high level, it indicates that the degree of IR is relatively high. In the HFD group, the serum hs-CRP level was significantly increased, while spinosin (20 and 40 mg/kg) and metformin (200 mg/kg) significantly decreased serum hs-CRP levels (Supplementary materials, Figure S2B).

***Spinosin reduced the sICAM-1, PAI-1, and ROS production in PA-stimulated HUVEC***

**Figure 2:**
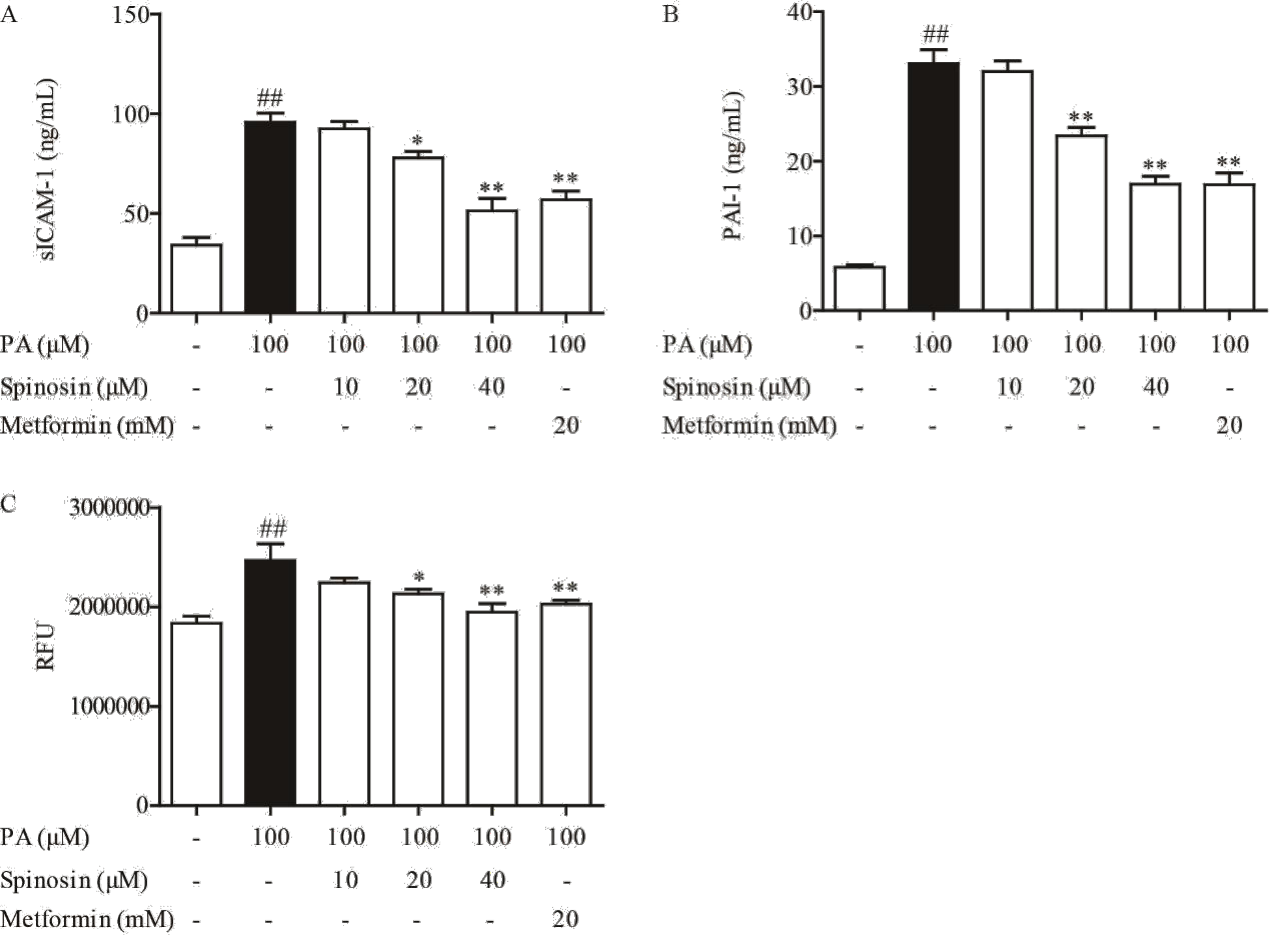
Effect of spinosin on levels of sICAM (A), PAI-1 (B), and ROS (C) in palmitic acid (PA)-induced HUVECs. HUVECs were exposed to a complete DMEM containing spinosin (10, 20, and 40 μM) or metformin (20 mM) for 30 min, then adding 100 μM PA for 24 hr, the levels of sICAM-1, PAI-1, and ROS were detected with respective kits. All data were expressed as the means ± SEM of three independent experiments. ##P<0.01 vs the group without any treatment; *P<0.05, **P<0.01 vs the group treated with PA

As already known, overproduction of ROS can be induced by excessive FFA in the circulation. In our study, exposing HUVEC cells to PA indeed induced ROS production. As shown in Figure 2, PA can increase the secretion of sICAM-1 and PAI-1, and promote the production of ROS in HUVECs. Spinosin (20 and 40 μM) suppressed the production of sICAM-1 and PAI-1 in the cell culture supernatant, and the also accumulation of ROS in the cell, suggesting potent antioxidant action of spinosin in the cell culture system.

***Spinosin suppresses IL-6 and TNF-a gene expression in PA treated HUVECs cells***

Previous research indicated PA stimulation resulted in phosphorylation of inhibitor of nuclear factor kappa-B kinase and nuclear factor kappa-B, then promoted the gene transcription and protein synthesis of a series of inflammatory factors. As shown in Supplementary materials’ Figure S3, PA promoted the expression of IL-6 and TNF-α in HUVECs. Spinosin concentration-dependent decreased the IL-6 and TNF-α production. These results suggest that spinosin suppressed the inflammatory response in the endothelium.

***Effects of spinosin on p38, ERK, and JNK levels in HUVEC cells induced by PA and insulin***

TNF-α blocked tyrosine phosphorylation of insulin receptors, then suppressed insulin signal transduction, and then activated the MAPKs (17). Activated MAPK family (p38, ERK, and JNK) can inhibit insulin receptor phosphorylation (8), and also induce more serious damage to insulin resistance by inducing inflammation. Activated JNK (18), ERK(19), and p38(20) were detected in insulin resistance. In PA and insulin-induced HUVECs, the total protein expression of p38, ERK and JNK remained unchanged, but the phosphorylation level was significantly increased. After spinosin treatment, phosphorylation of p38, ERK, and JNK proteins almost disappeared (Supplementary materials’ Figure S4).

***Effects of spinosin on the levels of IRS-1, PI3K, Akt, eNOS, and NO in HUVECs induced by PA and insulin***

**Figure 3:**
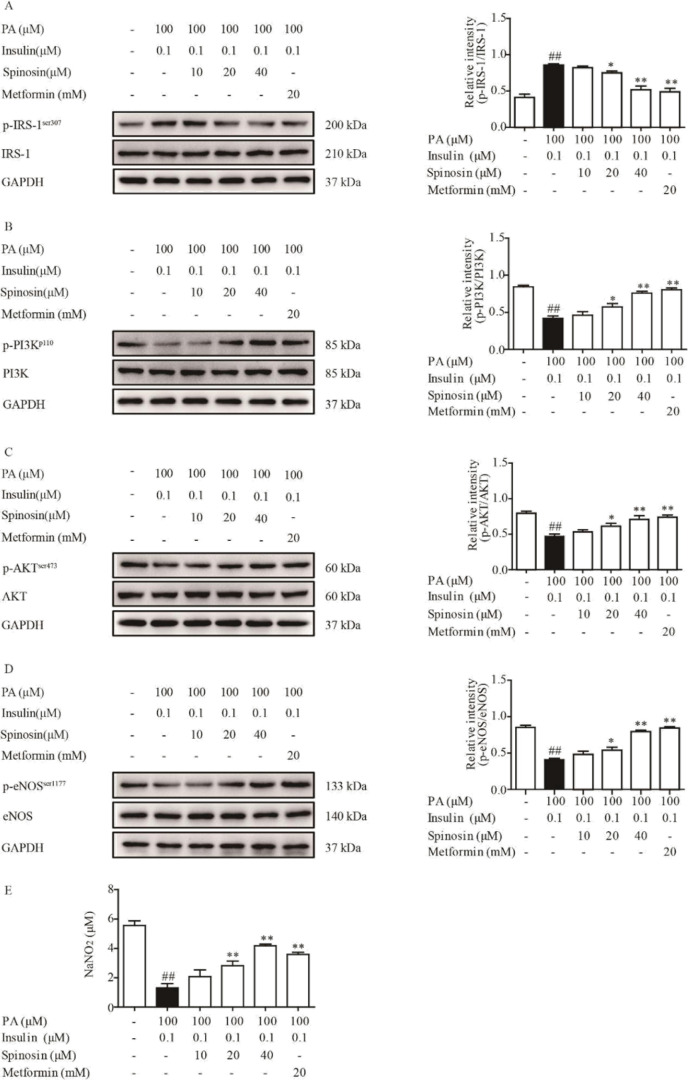
Effect of spinosin on activation of IRS-1/PI3K/AKT/eNOS signals and production of NO in palmitic acid (PA)-induced HUVECs. HUVECs were exposed to a complete DMEM containing spinosin (10, 20, and 40 μM) or metformin for 30 min, then adding 100 μM PA for 24 hr and 0.1 μM insulin for 30 min before the cells were collected. Finally, the cells were harvested and used for the assay of expressions of IRS-1, PI3K, AKT, eNOS, and IRS-1 (p-ser307) PI3K (p110), P-AKT (Ser473), and p-eNOS (Ser1177) by western blot analysis (A-D). (E) NO levels were detected with respective kits. All data were expressed as the means ± SEM of three independent experiments. ##*P*<0.01 vs the group without any treatment; *P<0.05, **P<0.01 vs the group treated with PA and insulin

**Figure 4:**
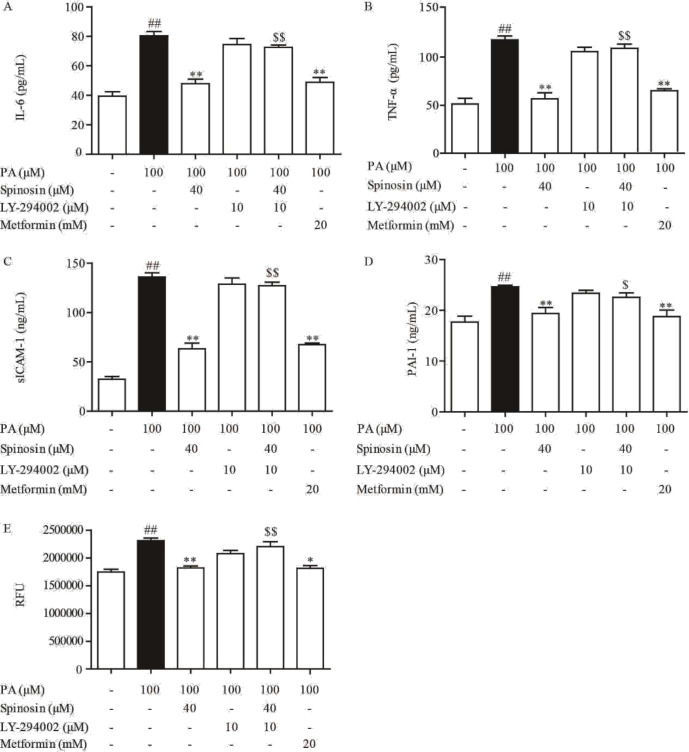
Participation of PI3K/AKT/eNOS signals in spinosin-mediated protection of endothelial cells. HUVECs were exposed to a complete DMEM containing spinosin (40 μM) or PI3K inhibitor LY-294002 or metformin for 30 min, then adding 100 μM palmitic acid (PA) for 24 hr, the levels of IL-6, TNF-α, sICAM-1, PAI-1, and ROS (A-E) were detected with respective kits. All data were expressed as the means ± SEM of three independent experiments. ##*P*<0.01 vs the group without any treatment; **P<0.01 vs the group treated with PA; $*P*<0.05 and $$*P*<0.01vs the group treated with spinosin

Phosphorylation of IRS-1 serine triggering inflammation plays an important role in impaired insulin signaling. In the presence of PA and insulin, IRS-1 was activated in HUVECs, but the total protein expression was not affected (Figure 3A). In the present study, spinosyn (20 and 40 μM) positively regulated IRS-1 function by inhibiting phosphorylation of IRS-1 at serine induced by PA, on the other hand, the restored IRS-1 tyrosine phosphorylation is in response to insulin. PA inhibited insulin-mediated tyrosine phosphorylation of IRS-1, in turn impairing downstream signaling of the PI3K/Akt/eNOS pathway. In addition, PA stimulation attenuated p-PI3K(p110) and p-Akt (Ser473) levels in response to insulin, but the inhibitory tendency was significantly reversed by spinosyn (Figures 3B and C). Activation of PI3K/Akt directly phosphorylates eNOS, which induces NO production in the endothelium. As expected, spinosyn restored insulin-mediated eNOS phosphorylation (p-eNOS(Ser1177) level) in the presence of PA (Figure 3D). In this process, the total protein expression of PI3K, Akt, and eNOS was not affected by PA, insulin, and spinosyn. IRS-1/PI3K/Akt/eNOS signaling pathway activation results in NO production, while insulin signaling impaired and blunted insulin action in PA-induced HUVEC, which decreases NO production. Spinosyn treatment restored the impairment of insulin-mediated NO production in PA-induced endothelial cells. We further demonstrated the direct effects of spinosyn on PI3K activity using LY-294002, a specific inhibitor of PI3K, LY-294002 significantly reduced the effect of spinosyn on IL-6, TNF-α, sICAM-1, PAI-1, and ROS production in PA treated HUVECs. (Figure 4).

***Spinosin showed vasodilation effect of ISO-pretreated rat aortae with PI3K dependent pathway***

**Figure 5:**
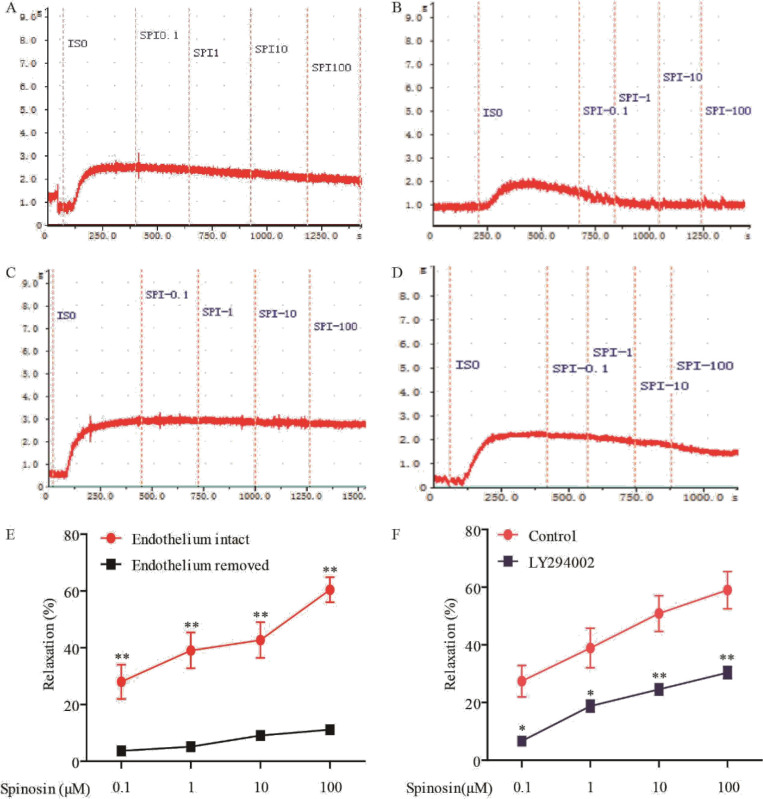
(A) Vasodilating effect of spinosin on isoprenaline pre-constriction in the vascular ring with intact endothelium. (B) Vasodilating effect of spinosin on isoprenaline pre-constriction in the vascular ring with damaged endothelium. (C) Statistical comparison of intact and removed endothelial vascular rings. All data were presented as mean ± SEM (n=6). **P<0.01 vs removed endothelial vascular rings. (D) Vasodilating effect of spinosin on isoprenaline pre-constriction in the vascular ring with intact endothelium. (E) Vasodilating effect of spinosin on isoprenaline pre-constriction in the vascular ring with PI3K inhibitor LY294002. (F) Statistical comparison of intact endothelial vascular rings with or without PI3K inhibitor LY294002. All data were presented as mean ± SEM (n=6). **P<0.01 vs control group

A feature of IR-associated endothelial dysfunction is the loss of endothelium-dependent vessel relaxation. In this work, spinosin at 0.1, 1, 10, and 100 μM had a vasodilation effect on the ISO pre-treated intact endothelial rat aortae, the maximum relaxation rate was 60.44% ± 10.75% (Figure 5A). However, spinosin at the same concentration does not affect endothelial-derived rat aortae, the maximum diastolic rate was 11.16% ± 45% (Figure 5B). As shown in Figure 5C, there is a statistically significant difference between the relaxation rate of spinosyn on the intact endothelial rat aortae and the endothelial-derived rat aortae (*P*<0.01), which indicates the vasodilation effect of spinosin on rat aortae is endothelium-dependent. Furthermore, we investigated the effect of PI3K inhibitor on the vasodilation of isolated rat aortic rings with intact endothelium by spinosin after ISO pre contraction. As shown in Figure 5 D-F, the maximum diastolic rate of the control group was 59.01% ± 15.76%. After the PI3K inhibitor LY294002 was added in advance, the relaxation effect of spinosin on intact endothelial aortic rings disappeared, and the maximum relaxation rate was 30.37% ± 5.06%. There was significant difference between the two groups (*P*<0.01), indicating that PI3K pathway was involved in the vasodilation induced by spinosin. As shown in Figure 5A, spinosin at 0.1°100 μM has a concentration-dependent relaxation effect on the ISO-induced contracted rat aortic rings with intact endothelium, the maximum relaxation rate was 60.44 ± 10.75%. However, spinosin does not affect the endothelial-derived aortic rings, the maximum relaxation rate was 11.16 ± 3.45% (Figure 5B). As shown in Figure 5C, spinosin had a statistically significant effect on the relaxation rate of intact endothelial and endothelial-derived rat aortae (*P*<0.01), so it can be inferred that the relaxation effect of spinosin on aortic rings is endothelium-dependent. Furthermore, we investigated the effect of PI3K inhibitor on the vasodilation of isolated rat aortic rings with intact endothelium by spinosin after ISO pre-contraction. As shown in Figure 5D-F, the maximum diastolic rate of the control group was 59.01 ± 15.76%. After LY294002 was pre-added, the relaxation effect of spinosin on endothelial intact aortic rings disappeared, and the maximum relaxation rate was 30.37 ± 5.06%, which was statistically significant between the two groups (*P*<0.01).

## Discussion

In this study, ICR mice were induced by HFD high-fat diet to establish an IR model. Compared with the HFD group, the weight gain, fasting serum glucose and insulin levels, oral glucose tolerance, and HOMA-IR of mice were significantly decreased by treatment with spinosin for 4 weeks. In addition, spinosin down-regulated the serum levels of FFA, T-Cho, TG, LDL-C, PAI-1, and hs CRP in insulin-resistant mice. The above results showed that spinosin could significantly improve IR in mice.

FFA lead excessive ROS production along with decreased antioxidant defenses resulting in oxidative stress, which plays a pivotal role in the initiation and development of diabetic endothelial dysfunction (21, 22). In addition, high level of FFA is associated with impaired endothelium-dependent vasodilatation and other cardiovascular disorders in clinical settings (23,24). In the present work, the major dietary saturated fatty acid, PA, was used to induce ROS production in endothelial cells, and stimulate IR. Spinosin treatment effectively reduced ROS, sICAM-1, and PAI-1 overproduction and facilitated insulin action on the HUVECs, demonstrating its protective effect against FFA stimulation.

ROS is well known to be associated with the inflammation process through various pathways (25,26). Spinosin is recognized as an antioxidant, indeed, it inhibited TNF and IL6 gene expression in PA treated HUVECs, which further indicates that spinosin can inhibit inflammation in endothelial cells by attenuating oxidative stress.

In the present study, PA stimulation blocked the functional interaction between insulin receptors and IRS-1 by inducing serine phosphorylation and down-regulated tyrosine phosphorylation in IRS-1 in response to insulin. Spinosin inhibited the reverse of serine/tyrosine phosphorylation induced by PA, and also suppressed the phosphorylation of MAPKs proteins, indicating spinosin restored IRS-1 function to inhibit the production and inflammatory reaction.

Inhibition of phosphorylation of IRS-1 at tyrosine led to the PI3K/Akt/eNOS signaling pathway. Spinosin restored phosphorylation of Akt and eNOS, indicating its protective effect on PI3K signaling against PA stimulation. The observed phenomenon was ascribed restoration of IRS-1 function after spinosin treatment. To testify the hypothesis, we studied the effects of spinosin on NO production regulated by PI3K activity with LY-294002. As expected, spinosin had little effect on IL-6, TNF-α, sICAM-1, PAI-1, and ROS production in LY-294002-treated cells. This result further suggested that spinosin improves Akt and eNOS phosphorylation by directly up-regulating PI3K activity. In the present study, spinosin was proven to increase insulin-stimulated NO production by endothelial cells through modulating IRS-1 function and PI3K signaling. Endothelial NO deficiency is considered the primary defect for the occurrence of IR and endothelial dysfunction, as well as endothelium-dependent vasodilation. PA impaired the insulin-dependent vasodilation of rat aortic rings, which was restored by spinosin in a concentration-dependent manner. The vasodilation effect of spinosin on rat aortic rings was also suppressed by PI3K inhibitor LY294002. Consistent with the effect on PI3K signaling, spinosin could improve insulin-mediated NO production and alleviate the endothelial dysfunction in IR.

## Conclusion

Spinosin ameliorated the phenotypes of IR and inhibited ROS production and sequential inflammation. Through these beneficial effects, spinosyn activated PI3K/Akt/eNOS signaling by restoring IRS-1 function, leading to insulin-mediated NO production in the endothelium. Our results demonstrated the possible mechanism of spinosin in the treatment of endothelial IR as well as cardiovascular complications in diabetes.

## Authors’ Contributions

CYG Designed the experiments; LY, JLZ, and ZFW Performed experiments and collected data; CYG, ZFW, and FF Discussed the results and strategy; LY and FF Supervised, directed, and managed the study; CYG Prepared the draft manuscript and visualization. FF Approved the final version to be published. 

## Conflicts of Interest

The authors declare that there are no conflicts of interest.
